# Asymptotic correlation functions and FFLO signature for the one-dimensional attractive spin-1/2 Fermi gas

**DOI:** 10.1016/j.nuclphysb.2011.07.007

**Published:** 2011-12-01

**Authors:** J.Y. Lee, X.W. Guan

**Affiliations:** Department of Theoretical Physics, Research School of Physics and Engineering, Australian National University, Canberra, ACT 0200, Australia

## Abstract

We investigate the long distance asymptotics of various correlation functions for the one-dimensional spin-1/2 Fermi gas with attractive interactions using the dressed charge formalism. In the spin polarized phase, these correlation functions exhibit spatial oscillations with a power-law decay whereby their critical exponents are found through conformal field theory. We show that spatial oscillations of the leading terms in the pair correlation function and the spin correlation function solely depend on $\Delta k_{F}$ and $2\Delta k_{F}$, respectively. Here $\Delta k_{F}=\pi (n_{↑}-n_{↓})$ denotes the mismatch between the Fermi surfaces of spin-up and spin-down fermions. Such spatial modulations are characteristics of a Fulde–Ferrell–Larkin–Ovchinnikov (FFLO) state. Our key observation is that backscattering among the Fermi points of bound pairs and unpaired fermions results in a one-dimensional analog of the FFLO state and displays a microscopic origin of the FFLO nature. Furthermore, we show that the pair correlation function in momentum space has a peak at the point of mismatch between both Fermi surfaces $k=\Delta k_{F}$, which has recently been observed in numerous numerical studies.

## 1 Introduction

Bardeen–Cooper–Schrieffer (BCS) theory was formulated over 50 years ago as a microscopic theory for superconductivity. One of the ingredients in BCS theory is pairing between electrons with opposite momenta and spins, i.e., matching between the Fermi energies of spin-up and spin-down electrons. In the phase where the system is partially polarized, Fermi energies of spin-up and spin-down electrons become unequal. This leads to a non-standard form of pairing which was predicted independently by Fulde and Ferrell [1], and Larkin and Ovchinnikov [2]. Fulde and Ferrell discovered that under a strong external field, superconducting electron pairs have nonzero pairing momentum and spin polarization. At about the same time, Larkin and Ovchinnikov suggested that the formation of pairs of electrons with different momenta, i.e., $\vec{k}$ and $-\vec{k}+\vec{q}$ where $\vec{q}\neq 0$, is energetically favored over pairs of electrons with opposite momenta, i.e., $\vec{k}$ and $-\vec{k}$, when the separation between Fermi surfaces is sufficiently large. Consequently, the density of spins and the superconducting order parameter become periodic functions of the spatial coordinates. This non-conventional superconducting state is known in literature as the Fulde–Ferrell–Larkin–Ovchinnikov (FFLO) state.

More recently, theoretical predictions of the existence of an FFLO state in one-dimensional (1D) interacting fermions [3], [4] have emerged by employment of various methods, such as Bethe ansatz (BA) [5], [6], density-matrix renormalization group (DMRG) [7], [8], [9], [10], [11], quantum Monte Carlo (QMC) [12], mean field theory [13], [14], [15], [16] and bosonization [17]. At finite magnetization, it was found by Feiguin and Heidrich-Meisner [7] that pair correlations for the attractive Hubbard model in a parabolic trapping potential has a power-law decay of the form $n^{\text{pair}}\propto cos(k_{\text{FFLO}}|x|)/{\left|x\right|}^{\alpha }$ and the momentum pair distribution has peaks at the mismatch of the Fermi surfaces $k_{\text{FFLO}}=\pi (n_{↑}-n_{↓})$. Wave numbers for the oscillations were numerically found as $\pi (n_{↑}-n_{↓})$ for the pair correlation function and as $2\pi (n_{↑}-n_{↓})$ for the density difference $\langle n_{↑}-n_{↓}\rangle$ [8]. The FFLO pairing wave number was also confirmed by the occurrence of a peak in the pair momentum distribution corresponding to the difference between the Fermi momenta of individual species [9], [12]. From mean field theory, it was demonstrated that the FFLO phase exists in the large-scale response of the Fermi gas [15] and even for temperatures up to $0.1T_{F}$ [14].

On the other hand, critical behavior of 1D many-body systems with linear dispersion in the vicinities of their Fermi points can be described by conformal field theory. Some time ago, the critical behavior of the Hubbard model with attractive interaction was investigated by Bogoliubov and Korepin [18], [19], [20], [21]. They showed that 1D superconductivity occurs when the average distance between electron pairs is larger than the average distance between individual electrons of these pairs. This means that the correlation function for the single particle Greenʼs function decays exponentially, i.e., $\langle \psi_{n,s}^{†}\psi_{1,s}\rangle \to e^{-n/\xi }$ with $\xi =v_{F}/\Delta$ and s=↑,↓, whereas the singlet pair correlation function decays as a power of distance, i.e., $\langle \psi_{n,↑}^{†}\psi_{n,↓}^{†}\psi_{1,↑}\psi_{1,↓}\rangle \to n^{-\theta }$. Here *Δ* is the energy gap, and the critical exponents *ξ* and *θ* are both greater than zero. This criterion is met when the external magnetic field is small, i.e., $H<H_{c}$. Once the external field exceeds the critical value, i.e., $H>H_{c}$, Cooper pairs are destroyed. Thus both of these correlation functions decay as a power of distance and the pairs lose their dominance, i.e., electrons become more or less independent of each other.

So far, theoretical confirmation of the FFLO state in 1D still relies on numerical evidence of spatial oscillations in the pair correlations. Despite key features of the T=0 phase diagram [5], [6], [22], [23], [24], [25] for the attractive Fermi gas were experimentally confirmed using finite temperature density profiles of trapped fermionic ^6^Li atoms [28], the unambiguous theoretical confirmation and experimental observation of FFLO pairing is still an open problem. As remarked in Ref. [9] that the 1D FFLO scenario proposed in Ref. [17] does not apply to 1D attractive fermions where quantum phase transition from the fully-paired phase into the spin polarized phase does not belong to commensurate–incommensurate university class, also see Refs. [22], [26]. For 1D attractive spin-1/2 fermions with polarization [3], [4], the low-energy physics of the homogeneous system is described by a two-component Tomonaga–Luttinger liquid (TLL) of bound pairs and excess unpaired fermions in the charge sector and ferromagnetic spin–spin interactions in the spin sector [27]. In this paper, we determine the critical behavior of the single particle Greenʼs function, pair correlation function and spin correlation function within the context of a TLL. We show that the long distance asymptotics of various correlation functions provide a microscopic origin of FFLO pairing for 1D attractive fermions.

This paper is organized as follows. We derive finite-size corrections for the ground state energy of the system in Section 2. In Section 3, we derive finite-size corrections for low-lying excitations and introduce the dressed charge formalism. Integral equations for each component of the dressed charge matrix is solved analytically in the strong coupling limit |c|≫1. In Section 4, we derive correlation functions for different operators and discuss the signature of FFLO pairing. Finally, conclusions and remarks are made in Section 5.

## 2 Ground state and finite-size corrections

We consider $N_{f}$ fermions with SU(2) spin symmetry in a 1D system of length *L* with periodic boundary conditions. The Hamiltonian for the spin-1/2 Fermi gas [3], [4] is given by

(1) $$H=-\sum_{j=1}^{N_{f}}\frac{\partial^{2}}{\partial x_{j}^{2}}+2c\sum_{1⩽j<k⩽N_{f}}\delta (x_{j}-x_{k}),$$

where c<0 is the attractive interaction strength. This model is one of the most important exactly solvable quantum many-body systems. In recent years, it has attracted considerable attention from theory [5], [6], [22], [23], [24], [25] and experiment [28] due to evidence of the FFLO state. Systems exhibiting novel phase transitions at T=0 are particularly useful in studying TLL physics [27] and the nature of the FFLO state.

The quasimomenta for unpaired fermions and bound pairs are given by $k_{j}$ and $\Lambda_{\alpha }\pm ic^{\prime }$ which satisfy the BA equations

(2) $$k_{j}L=2\pi I_{j}+\sum_{\alpha =1}^{N_{b}}2{tan}^{-1}\left(\frac{k_{j}-\Lambda_{\alpha }}{\left|c^{\prime }\right|}\right),$$

(3) $$2\Lambda_{\alpha }L=2\pi J_{\alpha }+\sum_{j=1}^{N_{u}}2{tan}^{-1}\left(\frac{\Lambda_{\alpha }-k_{j}}{\left|c^{\prime }\right|}\right)+\sum_{\beta =1}^{N_{b}}2{tan}^{-1}\left(\frac{\Lambda_{\alpha }-\Lambda_{\beta }}{2|c^{\prime }|}\right),$$

where quantum numbers $I_{j}$ and $J_{\alpha }$ are given by

(4) $$I_{j}\equiv \frac{N_{b}}{2}\;(mod\;1),\;J_{\alpha }\equiv \frac{N_{u}-N_{b}+1}{2}\;(mod\;1).$$

Here $c^{\prime }=c/2$, and $N_{u}$ and $N_{b}$ denote the number of unpaired fermions and bound pairs, respectively. The energy and momentum for this system reads

(5) $$E=\sum_{j=1}^{N_{u}}k_{j}^{2}+\sum_{\alpha =1}^{N_{b}}2\left(\Lambda_{\alpha }^{2}-{\left|c^{\prime }\right|}^{2}\right),\;P=\sum_{j=1}^{N_{u}}k_{j}+2\sum_{\alpha =1}^{N_{b}}\Lambda_{\alpha }.$$

We define monotonic increasing counting functions $z_{u}^{L}(k_{j}):=I_{j}/L$ and $z_{b}^{L}(\Lambda_{\alpha }):=J_{\alpha }/L$ and re-label the variables $k\to k_{u}$, $\lambda \to k_{b}$, $I_{j}\to I_{u,j}$ and $J_{\alpha }\to I_{b,\alpha }$ so that we can express the root densities in a general form as

(6) $$\rho_{u}^{L}(k_{u}):=\frac{d}{dk_{u}}z_{u}^{L}(k_{u})=\frac{1}{2\pi }-\frac{1}{L}\sum_{\alpha =1}^{N_{b}}a_{1}(k_{u}-k_{b,\alpha }),$$

(7) $$\rho_{b}^{L}(k_{b}):=\frac{d}{dk_{b}}z_{b}^{L}(k_{b})=\frac{1}{\pi }-\frac{1}{L}\sum_{j=1}^{N_{u}}a_{1}(k_{b}-k_{u,j})-\frac{1}{L}\sum_{\beta =1}^{N_{b}}a_{2}(k_{b}-k_{b,\beta }),$$

where $a_{n}(k)$ is defined by

(8) $$a_{n}(k)=\frac{1}{\pi }\frac{n|c^{\prime }|}{{\left(nc^{\prime }\right)}^{2}+k^{2}}.$$

Here $k_{\alpha ,j}$ (for $j=1,2,\ldots ,N_{\alpha }$ and α=u,b) denote the BA roots for unpaired fermions and bound pairs in the ground state.

Using the Euler–Maclaurin formula for contributions up to $O(1/L^{2})$ when L≫1, the finite-size corrections to the root densities can be written in the generic form as

(9) $$\rho_{\alpha }^{L}(k_{\alpha })=\rho_{\alpha }^{\left(0\right)}(k_{\alpha })+\sum_{\beta =u,b}\int_{-Q_{\beta }}^{Q_{\beta }}K_{\alpha \beta }(k_{\alpha }-k_{\beta })\rho_{\beta }^{L}(k_{\beta })\;dk_{\beta }+\frac{1}{24L^{2}}\sum_{\beta =u,b}\left[\frac{K_{\alpha \beta }^{\prime }(k_{\alpha }-Q_{\beta })}{\rho_{\beta }^{L}(Q_{\beta })}-\frac{K_{\alpha \beta }^{\prime }(k_{\alpha }+Q_{\beta })}{\rho_{\beta }^{L}(-Q_{\beta })}\right]\;(\alpha =u,b),$$

where

(10) $$\left(\begin{array}{c} \rho_{u}^{\left(0\right)}(k_{u})\\ \rho_{b}^{\left(0\right)}(k_{b}) \end{array}\right)=\left(\begin{array}{c} 1/2\pi \\ 1/\pi \end{array}\right),\;K(k)=\left(\begin{array}{cc} K_{uu}(k) & K_{ub}(k)\\ K_{bu}(k) & K_{bb}(k) \end{array}\right)=\left(\begin{array}{cc} 0 & -a_{1}(k)\\ -a_{1}(k) & -a_{2}(k) \end{array}\right).$$

Here, the Fermi points are denoted by $\pm Q_{\alpha }$. Notice that K(k) is a symmetric matrix.

In order to calculate finite-size corrections for the ground state and low energy excitations, we introduce the thermodynamic Bethe ansatz (TBA) [29], [30], which provides a powerful and elegant way to study the thermodynamics of 1D integrable systems. It becomes convenient to analyze phase transitions and low-lying excitations in the presence of external fields at zero temperature. In the thermodynamic limit, the grand partition function is $Z=\mathrm{tr}(e^{-H/T})=e^{-G/T}$, where the Gibbs free energy is given by $G=E-HM^{z}-\mu n-TS$, and is written in terms of the magnetization *H*, the chemical potential *μ* and the entropy *S* [30]. Equilibrium states satisfy the condition of minimizing the Gibbs free energy with respect to particle and hole densities for the charge and spin degrees of freedom (more details are given in Refs. [22], [30], [31], [32], [33]). At zero temperature, the ground state properties are determined by the dressed energy equations

(11) $$\varepsilon_{\alpha }(k_{\alpha })=\varepsilon_{\alpha }^{\left(0\right)}(k_{\alpha })+\sum_{\beta =u,b}\int_{-Q_{\beta }}^{Q_{\beta }}K_{\alpha \beta }(k_{\alpha }-k_{\beta })\varepsilon_{\beta }(k_{\beta })\;dk_{\beta }\;(\alpha =u,b),$$

where $\varepsilon_{\alpha }^{\left(0\right)}(k_{\alpha })$ are given by

(12) $$\left(\begin{array}{c} \varepsilon_{u}^{\left(0\right)}(k_{u})\\ \varepsilon_{b}^{\left(0\right)}(k_{b}) \end{array}\right)=\left(\begin{array}{c} k_{u}^{2}\\ 2k_{b}^{2}-{\left|c\right|}^{2}/2 \end{array}\right).$$

1D many-body systems are critical at T=0 and exhibit not only global scale invariance but local scale invariance too, i.e., conformal invariance. The conformal group is infinite dimensional and completely determines the conformal dimensions and correlation functions when the excitations are gapless [34]. Conformal invariance predicts that the energy per unit length has a universal finite-size scaling form that is characterized by the dimensionless number *C*, which is the central charge of the underlying Virasoro algebra [35], [36]. From the density distributions (9) and dressed energy equations (11), the finite-size corrections to the ground state energy is given by

(13) $$\varepsilon_{0}=\varepsilon_{0}^{\infty }-\frac{C\pi }{6L^{2}}\sum_{\alpha =u,b}v_{\alpha },$$

where C=1, and $v_{u}$ and $v_{b}$ are the velocities of unpaired fermions and bound pairs, respectively. They are defined as

(14) $$v_{\alpha }:=\pm \frac{d\varepsilon_{\alpha }(k_{\alpha })}{dp_{\alpha }(k_{\alpha })}{|}_{k_{\alpha }=\pm Q_{\alpha }}=\pm \frac{\varepsilon_{\alpha }^{\prime }(\pm Q_{\alpha })}{p_{\alpha }^{\prime }(Q_{\alpha })}=\pm \frac{\varepsilon_{\alpha }^{\prime }(\pm Q_{\alpha })}{2\pi \rho_{\alpha }(\pm Q_{\alpha })}\;(\alpha =u,b),$$

where prime denotes the derivative with respect to $k_{\alpha }$ and $p_{\alpha }(k_{\alpha })={lim}_{L\to \infty }\;2\pi z_{\alpha }^{L}(k_{\alpha })$. The term $\varepsilon_{0}^{\infty }$ represents the ground state energy in the thermodynamic limit, i.e., N,L→∞. In the strong coupling limit, exact expressions for the velocities can be found in Refs. [22], [37].

## 3 Low-lying excitations and dressed charge equations

Critical phenomena of critical systems are described by finite-size corrections for their low-lying excitations. The method we use to study correlation functions of the spin-1/2 Fermi gas with attractive interaction follows closely the method set out in Refs. [40], [41], [42], [43]. The conformal dimensions of two-point correlation functions can be calculated from the elements of the dressed charge matrix **Z**. Long distance asymptotics of various correlation functions are then examined through the dressed charge formalism at the T=0. Three types of low-lying excitations are considered in the calculations of finite-size corrections.

Type 1 excitation is characterized by moving a particle close to the right or left Fermi points outside the Fermi sea. It is equivalent to changing the quantum numbers $I_{\alpha ,j}$ close to $I_{\alpha }^{\pm }$ for unpaired fermions (α=u) and bound pairs (α=b). $I_{\alpha }^{\pm }$ characterize the Fermi points of each Fermi sea and are given by $I_{\alpha }^{+}=I_{\alpha }^{max}+1/2$ and $I_{\alpha }^{-}=I_{\alpha }^{min}-1/2$. The change in total momentum from Type 1 excitations is

(15) $$\Delta P=\frac{2\pi }{L}\sum_{\alpha =u,b}\left(N_{\alpha }^{+}-N_{\alpha }^{-}\right),$$

and the change in energy is

(16) $$\Delta E=\frac{2\pi }{L}\sum_{\alpha =u,b}\frac{\varepsilon_{\alpha }^{\prime }(Q_{\alpha }|Q^{\pm })}{p_{\alpha }^{\prime }(Q_{\alpha }|Q^{\pm })}\left(N_{\alpha }^{+}+N_{\alpha }^{-}\right)=\frac{2\pi }{L}\sum_{\alpha =u,b}v_{\alpha }\left(N_{\alpha }^{+}+N_{\alpha }^{-}\right).$$

Here $N_{\alpha }^{+}⩾0$ ($N_{\alpha }^{-}⩾0$) stems from the change in distribution of quantum numbers close to the right (left) Fermi points. This type of excitation is commonly known as particle–hole excitation.

Type 2 excitation arises from the change in total number of unpaired fermions or bound pairs. It is characterized by the change in quantum numbers

(17) $$N_{\alpha }=I_{\alpha }^{+}-I_{\alpha }^{-}\;(\alpha =u,b),$$

i.e., $\Delta N_{\alpha }=N_{\alpha }^{\text{excited}}-N_{\alpha }^{\text{ground}}$.

On the other hand, Type 3 excitation is caused by moving a particle from the left Fermi point to the right Fermi point and vice versa. This type of excitation is also known as backscattering. It is characterized by the quantum numbers

(18) $$\Delta D_{\alpha }=\frac{I_{\alpha }^{+}+I_{\alpha }^{-}}{2}\;(\alpha =u,b),$$

while leaving $\Delta N_{\alpha }$ unchanged.

All three types of excitations can be unified in the following form of the finite-size corrections for the energy and total momentum of the system

(19) ΔE=2πL(14(ΔN)tt(Z−1)VZ−1ΔN+(ΔD)tZVZtΔD+∑α=u,bvα(Nα++Nα−)),

(20) ΔP=2πL((ΔN)tΔD+NuΔDu+NbΔDb+∑α=u,bvα(Nα+−Nα−)).

Here we use the notations

(21) $$\Delta N=\left(\begin{array}{c} \Delta N_{u}\\ \Delta N_{b} \end{array}\right),\;\Delta D=\left(\begin{array}{c} \Delta D_{u}\\ \Delta D_{b} \end{array}\right),$$

$$V=\left(\begin{array}{cc} v_{u} & 0\\ 0 & v_{b} \end{array}\right),\;Z=\left(\begin{array}{cc} Z_{uu}(Q_{u}) & Z_{ub}(Q_{b})\\ Z_{bu}(Q_{u}) & Z_{bb}(Q_{b}) \end{array}\right).$$

The dressed charge equations are a set of four coupled integral equations that read

(22) $$Z_{uu}(k)=1-\int_{-Q_{b}}^{Q_{b}}a_{1}(k-\lambda )Z_{ub}(\lambda )\;d\lambda ,$$

(23) $$Z_{ub}(k)=-\int_{-Q_{u}}^{Q_{u}}a_{1}(k-\lambda )Z_{uu}(\lambda )\;d\lambda -\int_{-Q_{b}}^{Q_{b}}a_{2}(k-\lambda )Z_{ub}(\lambda )\;d\lambda ,$$

(24) $$Z_{bu}(k)=-\int_{-Q_{b}}^{Q_{b}}a_{1}(k-\lambda )Z_{bb}(\lambda )\;d\lambda ,$$

(25) $$Z_{bb}(k)=1-\int_{-Q_{u}}^{Q_{u}}a_{1}(k-\lambda )Z_{bu}(\lambda )\;d\lambda -\int_{-Q_{b}}^{Q_{b}}a_{2}(k-\lambda )Z_{bb}(\lambda )\;d\lambda .$$

Quantum numbers $\Delta D_{u}$ and $\Delta D_{b}$ (18) are chosen based on the conditions given in Eq. (4) and also on the conditions that $\Delta D_{u}\equiv \Delta N_{u}/2\;(mod\;1)$ and $\Delta D_{b}\equiv \Delta N_{b}/2\;(mod\;1)$. Combining both conditions together with the definition given in Eq. (18) yields

(26) $$\Delta D_{u}\equiv \frac{\Delta N_{u}+\Delta N_{b}}{2}\;(mod\;1),\;\Delta D_{b}\equiv \frac{\Delta N_{u}}{2}\;(mod\;1).$$

When the external magnetic field *H* is smaller than the critical field, spin excitations for this model are gapped. Once *H* exceeds this critical field, spin excitations become gapless and the system becomes conformally invariant. In this spin polarized phase, spin degrees of freedom are suppressed due to the ferromagnetic nature of excess unpaired fermions under a magnetic field. Therefore, bound pairs and excess unpaired fermions form two Fermi seas which can be described by a two-component TLL at low temperatures. Hence conformal invariance results in a universal finite-size scaling form of the energy shown in Eqs. (13), (19), and a universal form of the critical exponents of two-point correlation functions between primary fields $\langle O^{†}(x,t)O(x^{\prime },t^{\prime })\rangle$ which are determined by the finite-size corrections of the model. Multi-point correlation functions can be derived by taking the product of two-point correlation functions.

When T=0, the correlation functions of 1D systems decay as the power of distance, but when T>0 they decay exponentially. Following the standard calculations in Ref. [43], the conformal dimensions are given by

(27) $$2\Delta_{u}^{\pm }={\left(Z_{uu}\Delta D_{u}+Z_{bu}\Delta D_{b}\pm \frac{Z_{bb}\Delta N_{u}-Z_{ub}\Delta N_{b}}{2detZ}\right)}^{2}+2N_{u}^{\pm },$$

(28) $$2\Delta_{b}^{\pm }={\left(Z_{ub}\Delta D_{u}+Z_{bb}\Delta D_{b}\pm \frac{Z_{uu}\Delta N_{b}-Z_{bu}\Delta N_{u}}{2detZ}\right)}^{2}+2N_{b}^{\pm },$$

where $N_{\alpha }^{\pm }$ (α=u,b) characterize the descendent fields from the primary fields. General two-point correlation functions at T=0 take the form

(29) $$\langle O(x,t)O(0,0)\rangle =\frac{exp(-2i(N_{u}\Delta D_{u}+N_{b}\Delta D_{b})x)}{{\left(x-iv_{u}t\right)}^{2\Delta_{u}^{+}}{\left(x+iv_{u}t\right)}^{2\Delta_{u}^{-}}{\left(x-iv_{b}t\right)}^{2\Delta_{b}^{+}}{\left(x+iv_{b}t\right)}^{2\Delta_{b}^{-}}}.$$

The exponential oscillating term in the asymptotic behavior comes from Type 3 excitations, i.e., backscattering. Quantum numbers for the low-lying excitations completely determine the nature of the asymptotic behavior of these correlations. Here we are only concerned with the T=0 case.

The four dressed charge equations can be broken up into sets of two pairs. Eqs. (22), (23) constitute one pair, whilst Eqs. (24), (25) make up the other. Since we are interested in the strong coupling limit |c|≫1, both sets of equations can be solved iteratively up to accuracy 1/|c|. Let us consider the first set. Substituting Eq. (22) into Eq. (23) and iterating the terms give

(30) $$Z_{ub}(k)=-\int_{-Q_{u}}^{Q_{u}}\;d\lambda \;a_{1}(k-\lambda )+\int_{-Q_{b}}^{Q_{b}}\;d\lambda \;\int_{-Q_{u}}^{Q_{u}}\;d\lambda^{\prime }\;a_{2}(k-\lambda )a_{2}\left(\lambda -\lambda^{\prime }\right)-\int_{-Q_{u}}^{Q_{u}}\;d\lambda \;\int_{-Q_{b}}^{Q_{b}}\;d\lambda^{\prime }\;\int_{-Q_{u}}^{Q_{u}}\;d\lambda^{\prime \prime }\;a_{1}(k-\lambda )a_{1}\left(\lambda -\lambda^{\prime }\right)a_{1}\left(\lambda^{\prime }-\lambda^{\prime \prime }\right)+\cdots$$

The functions $a_{n}(k)$ have leading order 1/|c|, hence we can ignore all terms that have two or more multiples of $a_{n}(k)$. This procedure yields

$$Z_{ub}(Q_{b})\approx -\int_{-Q_{u}}^{Q_{u}}\;d\lambda \;a_{1}(Q_{b}-\lambda )\approx -\frac{4Q_{u}}{\pi |c|}.$$

Substituting Eq. (30) into Eq. (22), we obtain

(31) $$Z_{uu}(Q_{u})=1+\int_{-Q_{b}}^{Q_{b}}\;d\lambda \int_{-Q_{u}}^{Q_{u}}\;d\lambda^{\prime }\;a_{1}(Q_{u}-\lambda )a_{1}\left(\lambda -\lambda^{\prime }\right)+\cdots$$

(32) Zuu(Qu)≈1.

Next, we consider the second set of equations. Repeating the same arguments as before, Eq. (25) at the Fermi point $Q_{b}$ becomes

(33) $$Z_{bb}(Q_{b})=1-\int_{-Q_{b}}^{Q_{b}}\;d\lambda \;a_{2}(Q_{b}-\lambda )+\int_{-Q_{u}}^{Q_{u}}\;d\lambda \;\int_{-Q_{b}}^{Q_{b}}\;d\lambda^{\prime }\;a_{1}(Q_{b}-\lambda )a_{1}\left(\lambda -\lambda^{\prime }\right)+\int_{-Q_{b}}^{Q_{b}}\;d\lambda \;\int_{-Q_{b}}^{Q_{b}}\;d\lambda^{\prime }\;a_{2}(Q_{b}-\lambda )a_{2}\left(\lambda -\lambda^{\prime }\right)+\cdots$$

Zbb(Qb)≈1−2Qbπ|c|.

Eq. (24) at the Fermi point $Q_{u}$ then reads

(34) $$Z_{bu}(Q_{u})=-\int_{-Q_{b}}^{Q_{b}}\;d\lambda \;a_{1}(Q_{u}-\lambda )+\int_{-Q_{b}}^{Q_{b}}\;d\lambda \;\int_{-Q_{b}}^{Q_{b}}\;d\lambda^{\prime }\;a_{1}(Q_{u}-\lambda )a_{2}\left(\lambda -\lambda^{\prime }\right)+\cdots \approx -\frac{4Q_{b}}{\pi |c|}.$$

From Ref. [22], the Fermi points in the strongly attractive limit are given by

(35) $$Q_{u}\approx \pi n_{f}P\left(1+\frac{2(1-P)}{\left|\gamma \right|}\right),$$

(36) $$Q_{b}\approx \frac{\pi n_{f}(1-P)}{4}\left(1+\frac{\left(1-P\right)}{2|\gamma |}+\frac{2P}{\left|\gamma \right|}\right),$$

where $n_{f}=N_{f}/L$ is the density of fermions per unit length, $\gamma =c/n_{f}$ is the dimensionless interaction parameter and $P=(N_{↑}-N_{↓})/N_{f}=N_{u}/N_{f}$ is the polarization. Inserting these relations into the expressions for dressed charges, we obtain

(37) $$Z_{uu}(Q_{u})\approx 1,\;Z_{ub}(Q_{b})\approx -\frac{4P}{\left|\gamma \right|},$$

$$Z_{bu}(Q_{u})\approx -\frac{\left(1-P\right)}{\left|\gamma \right|},\;Z_{bb}(Q_{b})\approx 1-\frac{\left(1-P\right)}{2|\gamma |}.$$

**Fig. 1:**
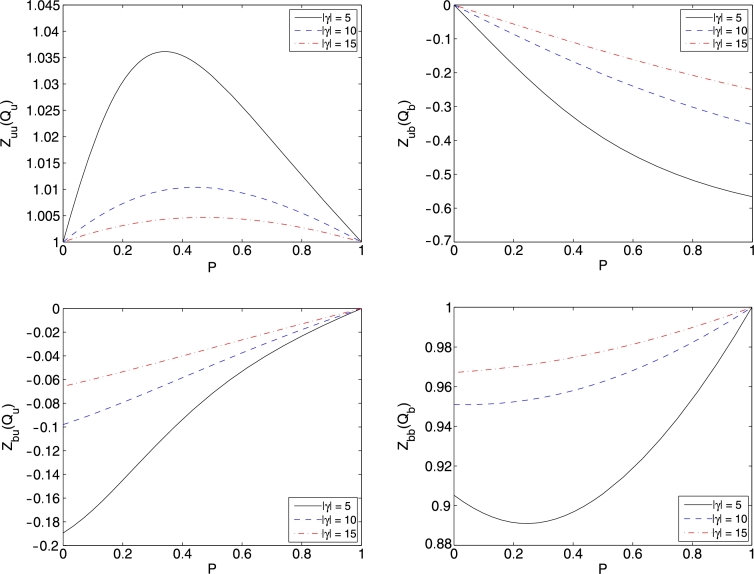
(Color online.) These figures show a plot of the dressed charges $Z_{uu}(Q_{u})$, $Z_{ub}(Q_{b})$, $Z_{bu}(Q_{u})$ and $Z_{bb}(Q_{b})$ versus polarization for different values of |*γ*|.

In Fig. 1, the dressed charges are numerically calculated and plotted against polarization for different values of interaction strength |γ|.

In the strong coupling limit, the external magnetic field *H* is related to the polarization as

(38) $$H\approx \frac{n^{2}{\left|\gamma \right|}^{2}}{2}+2\pi^{2}n^{2}P^{2}\left(1+\frac{4(1-P)}{\left|\gamma \right|}-\frac{4P}{3|\gamma |}\right)-\frac{\pi^{2}n^{2}{\left(1-P\right)}^{2}}{8}\left(1+\frac{4P}{\left|\gamma \right|}\right).$$

With this relation, we can evaluate the dressed charges for different values of *H*. From the expressions for the dressed charges in Eq. (37), the conformal dimensions $\Delta_{\alpha }^{\pm }$ in terms of polarization are given by

(39) $$2\Delta_{u}^{\pm }\approx {\left(\Delta D_{u}\pm \frac{\Delta N_{u}}{2}\right)}^{2}-\frac{8P}{\left|\gamma \right|}\left(\Delta D_{u}\pm \frac{\Delta N_{u}}{2}\right)\left(\Delta D_{b}\mp \frac{\Delta N_{b}}{2}\right)+2N_{u}^{\pm },$$

(40) $$2\Delta_{b}^{\pm }\approx \left(1-\frac{\left(1-P\right)}{\left|\gamma \right|}\right){\left(\Delta D_{b}\pm \frac{\Delta N_{b}}{2}\right)}^{2}-\left(\frac{8P}{\left|\gamma \right|}\Delta D_{u}\mp \frac{\left(1-P\right)}{\left|\gamma \right|}\Delta N_{u}\right)\left(\Delta D_{b}\pm \frac{\Delta N_{b}}{2}\right)+2N_{b}^{\pm }.$$

## 4 Correlation functions at zero temperature

Here we consider 4 types of correlation functions, namely the single particle Greenʼs function $G_{↑}(x,t)$, charge density correlation function $G_{nn}(x,t)$, spin correlation function $G^{z}(x,t)$, and pair correlation function $G_{p}(x,t)$. Each correlation function is derived based on the choice of $\Delta N_{u}$ and $\Delta N_{b}$.

The one particle Greenʼs function, which is also called the Fermi-field (FF) correlation function in some literature, decays exponentially when the external magnetic field is not strong enough to overcome the gap associated with the breaking of bound states [18], [19], [20], [21]. Once in the gapless phase, i.e., when $H_{c1}<H<H_{c2}$ where $H_{c1}$ and $H_{c2}$ are the critical fields mentioned in Ref. [22], every correlation function at zero temperature decays spatially as some form of power law [34], [35], [36], [38], [39]. $G_{↑}(x,t)$ is characterized by $\left(\Delta N_{u},\Delta N_{b})=(1,0\right)$ which in turn allows quantum numbers $\Delta D_{u}\in Z+1/2$ and $\Delta D_{b}\in Z+1/2$. The leading terms are then given by

(41) $$G_{↑}(x,t)=\langle \psi_{↑}^{†}(x,t)\psi_{↑}(0,0)\rangle \approx \frac{A_{↑,1}cos(\pi (n_{↑}-2n_{↓})x)}{{\left|x+iv_{u}t\right|}^{\theta_{1}}{\left|x+iv_{b}t\right|}^{\theta_{2}}}+\frac{A_{↑,2}cos(\pi n_{↓}x)}{{\left|x+iv_{u}t\right|}^{\theta_{3}}{\left|x+iv_{b}t\right|}^{\theta_{4}}},$$

where the critical exponents are given by

(42) $$\theta_{1}\approx 1+\frac{4P}{\left|\gamma \right|},\;\theta_{2}\approx \frac{1}{2}-\frac{\left(1-P\right)}{2|\gamma |}+\frac{4P}{\left|\gamma \right|},$$

$$\theta_{3}\approx 1-\frac{4P}{\left|\gamma \right|},\;\theta_{4}\approx \frac{1}{2}-\frac{\left(1-P\right)}{2|\gamma |}-\frac{4P}{\left|\gamma \right|}.$$

The first term in $G_{↑}(x,t)$ comes from $\left(\Delta D_{u},\Delta D_{b})=(1/2,-1/2\right)$ and the second term comes from $\left(\Delta D_{u},\Delta D_{b})=(1/2,1/2\right)$. The constants $A_{↑,1}$ and $A_{↑,2}$ cannot be derived from the finite-size corrections for low-lying excitations. Here we only aim to evaluate the long distance asymptotics of these correlation functions. Instead of using $N_{u}$ and $N_{b}$ in the oscillation term, we choose to use $n_{↑}=N_{↑}/L$ and $n_{↓}=N_{↓}/L$ to elucidate the imbalance in the densities of spin-up and spin-down fermions. Both sets of variables are related by the relations $N_{u}=N_{↑}-N_{↓}$ and $N_{s}=N_{↓}$.

Next we consider the charge density correlation function $G_{nn}(x,t)$ together with the spin correlation function $G^{z}(x,t)$. Both of these correlation functions are characterized by the set of quantum numbers $\left(\Delta N_{u},\Delta N_{b})=(0,0\right)$ which allows quantum numbers $\Delta D_{u}\in Z$ and $\Delta D_{b}\in Z$. The leading terms are given by

(43) $$G_{nn}(x,t)=\langle n(x,t)n(0,0)\rangle \approx n^{2}+\frac{A_{nn,1}cos(2\pi (n_{↑}-n_{↓})x)}{{\left|x+iv_{u}t\right|}^{\theta_{1}}}+\frac{A_{nn,2}cos(2\pi n_{↓}x)}{{\left|x+iv_{b}t\right|}^{\theta_{2}}}+\frac{A_{nn,3}cos(2\pi (n_{↑}-2n_{↓})x)}{{\left|x+iv_{u}t\right|}^{\theta_{3}}{\left|x+iv_{b}t\right|}^{\theta_{4}}},$$

(44) $$G^{z}(x,t)=\langle S^{z}(x,t)S^{z}(0,0)\rangle \approx {\left(m^{z}\right)}^{2}+\frac{A_{z,1}cos(2\pi (n_{↑}-n_{↓})x)}{{\left|x+iv_{u}t\right|}^{\theta_{1}}}+\frac{A_{z,2}cos(2\pi n_{↓}x)}{{\left|x+iv_{b}t\right|}^{\theta_{2}}}+\frac{A_{z,3}cos(2\pi (n_{↑}-2n_{↓})x)}{{\left|x+iv_{u}t\right|}^{\theta_{3}}{\left|x+iv_{b}t\right|}^{\theta_{4}}},$$

where the operators n(x,t) and $S^{z}(x,t)$ are given in terms of the fields as

(45) $$n(x,t)=\psi_{↑}^{†}(x,t)\psi_{↑}(x,t)+\psi_{↓}^{†}(x,t)\psi_{↓}(x,t),$$

(46) $$S^{z}(x,t)=\frac{1}{2}\left(\psi_{↑}^{†}(x,t)\psi_{↑}(x,t)-\psi_{↓}^{†}(x,t)\psi_{↓}(x,t)\right).$$

The critical exponents for asymptotic expressions of $G_{nn}(x,t)$ and $G^{z}(x,t)$ are

(47) $$\theta_{1}\approx 2,\;\theta_{2}\approx 2-\frac{2(1-P)}{\left|\gamma \right|},$$

$$\theta_{3}\approx 2+\frac{16P}{\left|\gamma \right|},\;\theta_{4}\approx 2-\frac{2(1-P)}{\left|\gamma \right|}+\frac{16P}{\left|\gamma \right|}.$$

The constant terms for $G_{nn}(x,t)$ and $G^{z}(x,t)$ come from the choice of quantum numbers $\left(\Delta D_{u},\Delta D_{b})=(0,0\right)$. The second, third and fourth terms arise from the choices (1,0), (0,1) and (−1,1), respectively.

Finally we consider the pair correlation function $G_{p}(x,t)$. This correlation function is characterized by the set of quantum numbers $\left(\Delta N_{u},\Delta N_{b})=(0,1\right)$ which allows quantum numbers $\Delta D_{u}\in Z+1/2$ and $\Delta D_{b}\in Z$. The leading terms are

(48) $$G_{p}(x,t)=\langle \psi_{↑}^{†}(x,t)\psi_{↓}^{†}(x,t)\psi_{↑}(0,0)\psi_{↓}(0,0)\rangle \approx \frac{A_{p,1}cos(\pi (n_{↑}-n_{↓})x)}{{\left|x+iv_{u}t\right|}^{\theta_{1}}{\left|x+iv_{b}t\right|}^{\theta_{2}}}+\frac{A_{p,2}cos(\pi (n_{↑}-3n_{↓})x)}{{\left|x+iv_{u}t\right|}^{\theta_{3}}{\left|x+iv_{b}t\right|}^{\theta_{4}}},$$

where the critical exponents are given by

(49) $$\theta_{1}\approx \frac{1}{2},\;\theta_{2}\approx \frac{1}{2}-\frac{\left(1-P\right)}{2|\gamma |},$$

$$\theta_{3}\approx \frac{1}{2}+\frac{8P}{\left|\gamma \right|},\;\theta_{4}\approx \frac{5}{2}-\frac{5(1-P)}{2|\gamma |}+\frac{8P}{\left|\gamma \right|}.$$

The first term in $G_{p}(x,t)$ arises from the choice of quantum numbers $\left(\Delta D_{u},\Delta D_{b})=(1/2,0\right)$, whilst the second term arises from the choice $\left(\Delta D_{u},\Delta D_{b})=(1/2,-1\right)$.

The leading order for the long distance asymptotics of the pair correlation function $G_{p}(x,t)$ oscillates with wave number $\Delta k_{F}$, where $\Delta k_{F}=\pi (n_{↑}-n_{↓})$. Meanwhile, the leading order for the spin correlation function $G^{z}(x,t)$, which can also be thought of as the correlation of the density difference between spin-up and spin-down fermions, oscillates twice as fast with wave number $2\Delta k_{F}$. The oscillations in $G_{p}(x,t)$ and $G^{z}(x,t)$ are caused by an imbalance in the densities of spin-up and spin-down fermions, i.e., $n_{↑}-n_{↓}$, which gives rise to a mismatch in Fermi surfaces between both species of fermions. These spatial oscillations share a similar signature as the Larkin–Ovchinnikov (LO) pairing phase [2]. Our findings of the wave numbers agree with those discovered through DMRG [7], [8], [9], QMC [12] and mean field theory [14]. Though from conformal field theory, we see clearly that the spatial oscillation terms in the pair and spin correlations are a consequence of Type 3 excitations, i.e., backscattering for bound pairs and unpaired fermions. A comparison between our results and the results from numerical methods in Refs. [7], [8], [9], [12] suggest that the coefficient $A_{p,1}$ is very much larger than the coefficient $A_{p,2}$ because the frequency of the oscillations in numerical studies of $G_{p}(x,t)$ is almost identical to $\pi (n_{↑}-n_{↓})$. This observation also applies to $G^{z}(x,t)$, where $A_{z,2}$ and $A_{z,3}$ are much smaller when compared with $A_{z,1}$.

The correlation functions in momentum space can be derived by taking the Fourier transform of their counterparts in position space. From Refs. [42], [43], the Fourier transform of equal-time correlation functions of the form

(50) $$g\left(x,t=0^{+}\right)=\frac{exp(ik_{0}x)}{{\left(x-i0\right)}^{2\Delta^{+}}{\left(x+i0\right)}^{2\Delta^{-}}},$$

where $\Delta^{\pm }=\Delta_{u}^{\pm }+\Delta_{b}^{\pm }$ is given by

(51) $$\tilde{g}(k\approx k_{0})∼{\left[\text{sign}(k-k_{0})\right]}^{2s}{\left|k-k_{0}\right|}^{\nu }.$$

The conformal spin of the operator is $s=\Delta^{+}-\Delta^{-}$ and the exponent *ν* is expressed in terms of the conformal dimensions as $\nu =2(\Delta^{+}+\Delta^{-})-1$.

Hence the equal time correlation functions near the singularities $k_{0}$ for the one particle Greenʼs function, charge density, spin and bound pairs are

(52) $${\tilde{G}}_{↑}(k)∼{\left[\text{sign}\left(k-\pi (n_{↑}-2n_{↓})\right)\right]}^{2s_{↑}}{\left|k-\pi (n_{↑}-2n_{↓})\right|}^{\nu_{↑}},$$

(53) $${\tilde{G}}_{nn}(k)∼{\left[\text{sign}\left(k-2\pi (n_{↑}-n_{↓})\right)\right]}^{2s_{nn}}{\left|k-2\pi (n_{↑}-n_{↓})\right|}^{\nu_{nn}},$$

(54) $${\tilde{G}}^{z}(k)∼{\left[\text{sign}\left(k-2\pi (n_{↑}-n_{↓})\right)\right]}^{2s_{z}}{\left|k-2\pi (n_{↑}-n_{↓})\right|}^{\nu_{z}},$$

(55) $${\tilde{G}}_{p}(k)∼{\left[\text{sign}\left(k-\pi (n_{↑}-n_{↓})\right)\right]}^{2s_{p}}{\left|k-\pi (n_{↑}-n_{↓})\right|}^{\nu_{p}},$$

where the exponents are given by

(56) $$2s_{↑}\approx 1+\frac{4P}{\left|\gamma \right|}-\frac{\left(1-P\right)}{\left|\gamma \right|},\;\nu_{↑}\approx \frac{1}{2}+\frac{8P}{\left|\gamma \right|}-\frac{\left(1-P\right)}{2|\gamma |},$$

(57) $$2s_{nn}=2s_{z}\approx 0,\;\nu_{nn}=\nu_{z}\approx 1,$$

(58) $$2s_{p}\approx 0,\;\nu_{p}\approx -\frac{\left(1-P\right)}{2|\gamma |}.$$

**Fig. 2:**
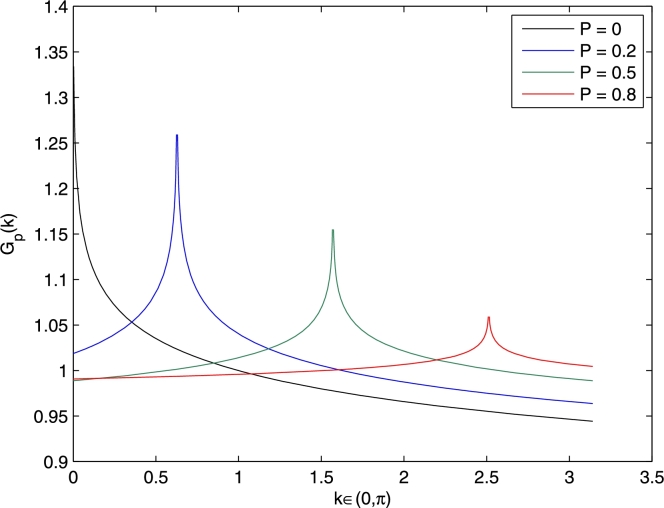
(Color online.) This figure shows a plot of the pair correlation function in momentum space ${\tilde{G}}_{p}(k)$ against *k* for different values of polarization *P* when |*γ*| = 10 and total linear density $n_{f}=1$. The location of the peaks are at *k* = 0, 0.2*π*, 0.5*π* and 0.8*π* when *P* = 0, 0.2, 0.5 and 0.8, respectively.

We would like to stress that the momentum space correlation functions derived in Eqs. (52), (53), (54), (55) are only accurate when the momenta *k* are within the proximity of the wave numbers $k_{0}$, i.e., when $k\approx k_{0}$. Fig. 2 plots ${\tilde{G}}_{p}(k)$ against *k* as polarization *P* varies between 0 to 0.8. This figure is in qualitative agreement with the ones given in Refs. [7], [9], [12]. We stress again that our plot is accurate only within the vicinity of the singularity, i.e., when *k* approaches $\pi (n_{↑}-n_{↓})$. We plotted ${\tilde{G}}_{p}(k)$ for the entire domain k∈(0,π) so that readers can visualize the curves more easily.

## 5 Conclusion

In conclusion, we investigated various zero-temperature correlation functions for the spin-1/2 Fermi gas with attractive interaction. We derived the finite-size corrections for ground state and low-lying excitations of the model. Using conformal field theory, critical exponents of the correlation functions were given in terms of polarization and interaction strength. We found that the leading terms of the pair correlation function and the spin correlation function oscillate with frequencies $\pi (n_{↑}-n_{↓})$ and $2\pi (n_{↑}-n_{↓})$, respectively. We also found that backscattering between the Fermi points of bound pairs and unpaired fermions results in a 1D analog of the FFLO state and displays a microscopic origin of the FFLO nature. Furthermore, we showed that there is a peak in the pair correlation function in momentum space at $k=\pi (n_{↑}-n_{↓})$ which confirms the oscillation frequency.

In the spin polarized phase, these correlation functions exhibit spatial oscillations with a power-law decay. This critical behavior can be viewed as an analogy to long range order in 1D, i.e., the power law decay of the pair correlation function which is regarded as evidence of a superconducting/superfluid state. We also like to mention that from the dressed charge formalism, the asymptotic behavior of the correlation functions derived in this paper can be numerically obtained with high accuracy for arbitrary interaction strength. Additionally, by considering weakly perturbed inter-tube interactions or inter-lattice interactions (1D fermionic Hubbard model), quasi-1D correlations in the spin polarized phase can be calculated from perturbation theory [19]. This provides a promising opportunity to estimate the critical temperature for high-$T_{c}$ superconductors/superfluids by studying 1D to 3D trapped cold atoms.
